# Supplementary material for: A rapid rate of sex-chromosome turnover and non-random transitions in true frogs
Source: Nat Commun. 2018 Oct 5;9:4088. doi: 10.1038/s41467-018-06517-2 (PMC6173717; doi:10.1038/s41467-018-06517-2)
Supplement: Supplementary file 1 — Supplementary Information [file 41467_2018_6517_MOESM1_ESM.pdf]

Supplementary Information

A rapid rate of sex-chromosome turnover and non-random transitions in true frogs

Daniel L. Jeffries*, Guillaume Lavanchy*, Roberto Sermier, Michael J. Sredl, Ikuo Miura, Amaël Borzée, Lisa N. Barrow, Daniele Canestrelli, Pierre-André Crochet, Christophe Dufresnes, Jinzhong Fu, Wen-Juan Ma, Constantino Macías Garcia, Karim Ghali, Alfredo G. Nicieza, Ryan P. O'Donnell, Nicolas Rodrigues, Antonio Romano, Íñigo Martínez-Solano, Ilona Stepanyan, Silvia Zumbach, Alan Brelsford, Nicolas Perrin

*co-first authors

Supplementary Note 1

Validating sex linkage in RADseq data

When using next-generation sequencing data to screen for and locate sex-linked markers, a primary concern is the presence of false positives. Thus, to validate signals for sex linkage across loci, two questions must be asked; are genotypes accurate, and is the signal at these loci artifactual? The former concerns the library preparation, sequencing and analyses steps, whereas the latter could also be caused by several aspects of study design (e.g. sample number, relatedness) if they are biased towards one sex. Many studies have accounted for this via the use of PCR either using sex-specific primers[1–5], or primers which amplify in both sexes, followed by amplicon sequencing[6] or digestion (PCR-RFLP)[5]. These approaches address both questions above; they confirm genotype, and they also allow for tests of sex linkage at given loci in a larger, independent sample. Unfortunately, the optimisation of PCR primers becomes prohibitively labour intensive for large-scale analyses like the present study. Furthermore, it is already known in several frog systems, that sex linkage along a sex chromosome can differ between, and sometimes even within the same population[7,8]. Therefore, the practice of using an independent sample set to confirm sex linkage of a given locus is not tenable in this system.

Instead, we implemented a bioinformatic validation of putatively sex-linked markers using a permutation approach which shuffles the assignments of sex across samples and compares the number of sex-linked markers found in each case with the number found using true sex assignments. False positive rate has been previously shown to rise as the number of samples

decreases and as the skew in the number of each sex increases[1,9]. As the overall sample number and the number of males and females remain constant in our permutation approach, both of these factors are accounted for in the generation of the null distribution. This approach also accounts for any unforeseen data characteristics that might also inflate false positive rate, e.g. paralogous loci which can be over-merged during STACKS processing, or population structure (as long as it is not the result of sex-biased dispersal). It should be noted that if a dataset passes this test (i.e. the number of sex linked markers identified with real sex assignments is outside of the 99th percentile of the null distribution), it does not imply that all markers in the set of putatively sex-linked markers are actually sex linked; false positives may still be present. However, an additional benefit of this method is that the mean of the null distribution also gives an indication of how many false positives can be expected. For instance, in *R. dalmatina*, 186 putatively sex linked loci were identified but the mean of the null distribution for this dataset indicates that approximately 25 of these are false positives.

Supplementary Note 2

Simultaneous XY and ZW signal on the same chromosome in RADseq datasets

Of the 20 species for which we analysed RADseq data, 12 had sex-linked marker sets that passed these sex-permutation validation tests for at least one method (Supplementary Fig. 1, Supplementary Table 1). In four species however (see *R. montezumae, R. arvalis, R. italica* and *R. kukunoris*, Supplementary Fig. 1), sets of sex-linked markers supporting both XY and ZW systems passed validation in the same species. In these cases, where sex-linked markers could be mapped to the reference genome, we found that XY and ZW markers mapped to the same chromosome. The two most obvious of these cases, *R. montezumae* and *R. italica* (each represented by clutches of full siblings) are shown in Supplementary Fig. 2. As it is biologically implausible that both XY and ZW sex-determination systems simultaneously exist on the same chromosome, we hypothesized that the ZW signal actually resulted from alleles that were specific to the X chromosome. Below we outline how such loci can result in false ZW signal in an XY species, and describe the analyses performed here, and independent lines of evidence that allow us to be confident that these four species are indeed truly XY.

There are two ways in which X-specific markers can create false ZW signal (but note, in all cases, the same would theoretically be true of the Z-specific markers creating false XY signal).

1) In data from a single family, it is possible to find alleles that are specific to the paternal X chromosome, due to standing variation on the X copies in the population from which the family comes. As the paternal X chromosome must be passed to all daughters, and no sons, all daughters in the family will be heterozygous, and sons homozygous at these loci – a pattern consistent with ZW heterogamety (Supplementary Fig. 2c,d). Importantly however, ZW expectations at these loci are violated in the parents, whereby the father would be heterozgygous and the mother homozygous – the reverse of a true ZW system (Supplementary Fig. 2c,d). Thus, to distinguish between ZW signal and X-specific signal in family data, it is necessary to examine the parental genotypes at such loci.

2). The second way in which X-specific markers can produce ZW-like signal is if there is a null allele at a RAD locus on the Y chromosome (via loss of a restriction enzyme cut site). This can occur in both family and population data. In such a case, females will posses two copies of the RAD locus, and due to standing variation on the X in the population, have the ability to be heterozygous. Males however, being hemizygous, possess only the X copy of the

locus, which will be interpreted as homozygous in RADseq data analyses. This would provide the female-heterozygous, male-homozygous pattern expected under a ZW system. In this case however, it is possible to test for a significant reduction in coverage at such loci in males, relative to the females (given a sufficient number of such loci exist). However, in family data, if many loci with paternal-X specific alleles also exist, they will likely mask the coverage difference between males and females at ZW-like markers as they are diploid in both sexes.

Below we describe analyses and independent lines of evidence for the four species that exhibit both XY- and ZW-like signal.

R. montezumae.

This dataset consists of two clutches of offspring, one for which no parental samples were avaiable and one for which the mother but not the father were available . In the latter family we identified 1867 XY markers and 1326 ZW markers, which mapped to the same chromosome of the reference (Supplementary Fig. 2a,b). As data was available for the mother, we were able to check her genotype at the ZW-like loci. Under a ZW heterogametic system, she would be expected to be heterozygous for these loci, however it is clear that she is homozygous at all of them, strongly supporting the hypothesis that these are in fact loci with paternal-X specific alleles. We can thus be confident that this species has an XY system of heterogamety.

R. arvalis.

This dataset comes from a single population of sampled adults. We identified 314 XY loci and 8 ZW linked loci. This dataset was already published by Brelsford et al. (2017), where they showed that one of these loci has lower coverage in males, as expected if it is hemizygous and specific to the X, and they also performed PCR validation to confirm that this species was indeed XY. Coverage analyses in the ZW-like markers found here suffers from low number of markers, but based on the analyses from the previous paper, we can be confident that this species is indeed XY.

R. kukunoris (Nanping)

This dataset comes from a single population of sampled adults. We identified 350 XY linked

markers, and 17 ZW-like markers. We performed coverage depth comparisons between males and females at these 17 ZW-like markers and found that average male coverage is significantly lower than in females (Welch's t-test, $t = -2.34$, $p = 0.48$, $df = 40$), whereas no difference was found at XY markers or autosomes. This supports the theory that these markers are X-specific and hemizygous in males, pointing to an XY system of heterogamety.

R. italica.

This dataset consists of a single clutch of offspring, but no parental data was available. We identified 1058 XY loci and 221 ZW loci, which mapped to the same chromosome of the reference (Supplementary Fig 2. b). Unfortunately, lacking data from either parent meant that we could not directly test for paternal-X specific alleles. We did test for lower male coverage at these loci, in an attempt to identify hemizygous (X-specific) loci, however no significant difference between male and female coverage was found (Welch's t-test, $t = -1.04$, $p = 0.59$).

There is however, a separate line of evidence which points to *R. italica* having an XY sytem of heterogamety. It is known that Anurans across several families, including Ranidae[10,11] and Hylidae[12] exhibit strong heterochiasmy whereby males undergo fewer recombination events during meioses and chiasmata are located predominantly at the tips of chromosomes. In an XY system, this means that almost the entire length of Y chromosomes do not recombine, which results in the distribution of sex linked markers shown in the Fig. 3 of the present manuscript. Indeed the fact that all five species for which sex linked markers could be aligned to the reference show this distribution is consistent with strong heterochiasmy being a common feature among Ranids. The Haldane-Huxley rule posits that achiasmy (i.e. total lack of recombination in one sex) follows the system of heterogamety, however this is not the case in heterochiasmatic species. In *G. rugosa* for example several independent XY→ZW transitions have occurred and we know that the ancestral pattern of heterochiasmy is retained (i.e. much reduced recombination in males), despite the transition in heterogamety[11]. Indeed current thinking surrounding the evolution of heterochiasmy is that it is not constrained by the system of heterogamety itself, but selected for over time[13], implying that it would take some time to "catch up" after a change in heterogamety. We can therefore expect that if a ZW transition occurred in a species studied here, the new Z and W chromosomes would continue to recombine along most of their length in females. In this case, markers which were truly ZW linked should cluster in the region immediately around the sex determiner or any localised regions where recombination arrest has been selected for in the short time since the

transition (i.e. following the canonical model of sex chromosome evolution). However, the ZW-markers identified in *R. italica* here are spread across almost the entire length of the chromosome, as expected from XY linked markers. Although anecdotal, we believe this makes it very unlikely that the ZW-like markers in this species represent a recent transition to ZW, and instead strongly support an XY system of heterogamety in this species.

There is one more line of evidence that supports the hypothesis that these species are XY, in each species above, that is there are many more XY-like markers than ZW-like markers. This is in line with the expectation that more Y-specific alleles should accumulate than X-specific alleles. Thus, taking together all of the above, we are confident that in the cases where both XY-like and ZW-like markers pass validation, the true system of sex determination is XY.

Supplementary Note 3

Testing which dataset parameters predict success of screens for sex-linked markers.

Before screening our new data for sex-linked markers, we performed preliminary analyses on an already published dataset from *R. arvalis*[9] to identify which dataset characteristics predict the success of screens for sex linkage. Interestingly, the factor which had the most pronounced effect on the success of sex-linked marker screens was the inclusion of two Y haplotypes (Hap1, Hap2) in the *R. arvalis* sample (Subsample 2 in Supplementary Fig. 3b). For this subsample ($6M^{Hap1}$, $6M^{Hap2}$, 12F) no XY sex-linked markers were reliably identified. The occurrence of multiple Y haplotypes within a same population has been documented in other frog populations[8,14], and might thus have acted as a significant obstacle to sex-linked marker identification in our datasets. This *R. arvalis* dataset was quite robust to reducing the number of samples overall (when sex ratios remained equal). From sample sizes between 19 Males (M), 19 Females (F) down to 13M, 13F, false positive rate was relatively low and the number of sex linked markers identified remained relatively constant. However the number of false positives increased with each round of downsampling and became considerable at sample sizes of 9M, 9F and below. Although sex-linked markers could still be identified down to samples as low as 7M, 7F, the false positive distributions suggest that these marker sets will contain a large number of false positives. In such cases validation of these markers would be imperative, e.g. via alignment to a genome (to check for clustering on a single chromosome) or PCR. The number of false positives drastically rose at 5M, 5F to the point where the real sex-linked marker dataset would not pass our filter for reliability, and the system of heterogamety could not be called. Thus, sample size is one major predictor for the success of sex-linked marker screens (Supplementary Fig. 9c). Indeed, in most of our datasets for which no sex-linked markers could be reliably found, sample sizes were relatively small (e.g. *R. berlandieri* (11M, 9F), *R. yavapaiensis* (9M, 10F), *P. saharicus* (14M, 7F), *R. uenoi* (8M, 10F), *R. macrocnemis* (6M, 8F). However, sex-linked markers could be confidently identified in several datasets of similar or smaller sample sizes, suggesting that this is not the only predictor for the success of such screens. Lastly, we found firstly that sex-linked marker screens were also quite robust to skews between male and female sample numbers. However, although false positive rate increased during all downsampling, skewing samples towards males (e) resulted in a higher number of putatively sex-linked markers found, with roughly the same false positive rate as the same skew towards females (d). This is likely because the condition that 90% of females (homogametic sex) must be homozygous for a sex-linked

locus is a very strong filter compared to the 50% heterozygosity in males. These results would therefore suggest that, given a limitation on sample number (i.e. space on a sequencer), investing more data into the heterogametic sex will likely yield better results than the other way around.

Supplementary Note 4

Making linkage maps, anchoring and ordering *R. temporaria* genome scaffolds.

Methods

In order to increase the number of RADtag markers that could be placed on the *X. tropicalis* reference genome, we undertook to anchor as many scaffolds as possible from our existing highly fragmented *R. temporaria* genome assembly[15] to the *X. tropicalis* assembly, so that they could in turn be used to infer the location of sex-linked RADtags from Ranid species. To do this, we first produced 12 separate sex-specific linkage maps using RADseq for 6 *R. temporaria* families from Tvedöra, Southern Sweden, each ranging between sizes of 60-90 offspring, plus parents (Supplementary Table 1). Linkage mapping was performed using MSTmap[16] (as implemented in the R package ASmap[17]). We used the cross type 'DH' and the p-value (which controls the sensitivity of the mapping) was optimized separately for each family, with the final value taken to be that which maximized the number of markers in the largest 13 linkage groups (as *R. temporaria* has 13 chromosomes). Second, we aligned linkage mapped RADtags from male maps to the *R. temporaria* assembly in order to assign scaffolds to linkage groups. We used only the male linkage maps from each family as the extremely low recombination rates in *R. temporaria* males (across all chromosomes)[15] allows many more markers to be assigned to linkage groups than in female maps. Third, we extracted a 4kb region of the *R. temporaria* scaffolds around each linkage map marker and aligned these to the *Nanorana parkeri* reference genome[18]. This process was then repeated, aligning 4kb segments of the mapped *N. parkeri* scaffolds to the *X. tropicalis* genome. Using this method, which has been shown to be effective at overcoming the large divergence between the species for which these genomic resources are available[9], any *R. temporaria* scaffold which had been assigned to that linkage group could also be assigned to a *X. tropicals* chromosome. Note that synteny is known to be highly conserved between *N. parkeri*, *X. tropicalis* and Ranidae[19,20]. This mapping procedure is summarized in Supplementary Fig. 4. These scaffolds are henceforth referred to as chromosome-assigned and were used in order to infer the chromosome to which sex-linked markers belong.

Although the limited male recombination makes the maps ideal for assigning markers to chromosomes, the order of markers within linkage groups is not informative, as almost all will have the same position. We therefore reanalyzed the data from our 6 *R. temporaria* families using LepMap3[21] in order to create a single combined map for females across all

families. We then repeated the three step mapping procedure outlined above to anchor scaffolds to their homologous position in the *X. tropicalis* genome. These scaffolds are henceforth referred to as chromosome-ordered scaffolds, and were used to infer the relative position of sex-linked markers within the sex chromosomes of species. All alignments mentioned above were performed using blastn v2.3.0[22], with hits only retained if their e-value was below 1×10^{-20} and, in the case of multiple matches, at least five orders of magnitude lower than that of the next best hit.

Results

Due to the significantly lower recombination rate in males, all male maps made in MSTmap contained more SNPs and correctly identified 13 linkage groups, corresponding to the 13 chromosomes in *R. temporaria.* However, in several of the female maps, higher or lower numbers of linkage groups were identified, indicating that there was a lack of power in these maps to confidently identify chromosomal linkage blocks. In total, the male maps contained 15,313 polymorphic RADtag markers (Supplementary Table 2). These markers mapped to 12,467 *R. temporaria* scaffolds. Of these, 5,638 aligned to 4,208 *N. parkeri* scaffolds, and of these, 1,782 could be aligned to a chromosome of the *X. tropicalis* genome. Importantly the 6,829 linkage-map-assigned *R. temporaria* scaffolds which could not be aligned to the *N. parkeri* or *X. tropicalis* genomes could still be assigned to a chromosome due to their linkage with successfully aligned scaffolds. In total this approach allowed us to assign 408.14 Mb (9.07 %) of the *R. temporaria* assembly to *X. tropicalis* chromosomes, however their order within these chromosomes was unknown.

The LepMap3 linkage map contained 10,853 RADtag markers, confidently identified 13 linkage groups as expected (Supplementary Fig. 10) and showed the typical drastic difference between male and female recombination patterns previously observed for this species. The mapped RADtags aligned to 3,397 scaffolds and allowed us to order 328 Mb (7.31 %) of the *R. temporaria* genome. These scaffolds are referred to in the main text as “ordered scaffolds”.

Supplementary Note 5

Calculating the rate of transitions

In this section we propose an approach for calculating an approximate transition rate across the phylogeny, which we envisage could be used to compare rates across different studies. To do this, one could simply count the number of turnovers and then divide by the sum of the branch lengths across the phylogeny. However this approach does not account for differences in taxon reporting, for example some studies (present study included) will include species in the phylogeny for which nothing is known about the character state of interest, whereas others will include only species where states are known. Using this approach, branches for species with unknown states will still be incorporated into the rate calculation, thus biasing estimates towards lower rates (as no transitions can be inferred on such branches).

Instead, we opted for a method that considers only the relevant parts of each tree: we perform all possible pairwise comparisons between species of interest on the tree, counting the evolutionary distance (i.e. branch lengths) and the number of turnovers observed between them.

We first implemented this approach by calculating a transition rate for each species pair independently and averaging across all rates at the end as follows:

$$\sum_n \cdot \frac{T}{d}$$

where n is the number of species pairs comparisons, T the number of turnover events that occurred along the branches between both species in each comparison and their MRCA, and d is the evolutionary distance between these two species, measured as the sum of branch lengths (in million years).

However, this approach allows the overall rate to be heavily influenced by comparisons between closely related species (i.e. short branch lengths). In light of this we opted for an approach where, instead of calculating a rate per pairwise comparison and averaging across the tree, we simply divide the total number of transitions encountered between all pairs by the total evolutionary distance from all pairwise comparisons. Formally this is:

$$\frac{\sum_n T}{\sum_n d}$$

It should be noted that this method is proposed only as a way to compare approximate transition rates between studies. It does not account for differences in taxon resolution and it will suffer from biased taxon reporting, where species are only included if a turnover has been observed on their branch. Incidentally, we encourage researchers to include all taxa when undertaking comparative studies on sex chromosome transitions, as even a lack of a transition is biologically meaningful.

Calculating the rate of transitions for Ranidae

We applied the above approach to our phylogeny. *P. porosus* was initially not included in our phylogeny, but the age of its MRCA with *P. nigromaculatus* was needed for our analysis. We therefore used the estimated divergence time of 9.9 M years taken from timetree.org[23]. For species in which different populations use different chromosome pairs for sex determination, we split these branches, assuming 2My (i.e. intraspecific levels) of divergence between all populations. This divergence is likely overestimated for most species (but see Sumida & Ogata[24]). However, no dated phylogeography was available for any of the concerned species, and underestimating divergence would have led to overestimating turnover rate. Note that our chosen approach reduces the impact of this assumption on the overall rate, as comparisons between closely related taxa carry less weight in the average rate calculation. All analyses were conducted in R using phytools and custom scripts, which can be accessed following instructions in the Data availability section of this paper.

The estimated rate of transitions across our phylogeny was 0.02 turnovers per My. This translates into 1 turnover for every 50 M years of independent evolutionary time (i.e. we would expect to see 1 turnover between two species which were 25 M years diverged from one another). Unfortunately, we are unable to formally compare this rate with other systems as this would require both reliable information on the chromosome pair that determines sex for a wide array of species within a clade, and a robust dated phylogeny of these species. Frequent turnover of sex-determination systems or sex chromosomes have been described in

many taxa. In geckos, Gamble et al.[2] have described a high rate of transitions from temperature to genetic sex-determination, but comparing sex chromosome turnovers with sex-determination system transitions is not informative. Several different sex chromosomes have been described among species of Salmonid fishes (reviewed in Sutherland et al.[25]). However, no study has attempted to summarise these findings and to place them in a phylogenetic framework so far. Finally, several sex-chromosome turnovers have been described in *Oryzias* fishes. Myosho et al.[26] have documented the sex chromosome in several species and summarised the available literature. They highlighted some turnovers, but their analysis is not exhaustive and they did not conduct an ancestral state reconstruction analysis, making it impossible to estimate on which branches turnovers occurred. Additionally, the age of the *Oryzias* radiation is not yet settled, with a threefold difference between studies[27,28], which would vastly influence turnover rate estimates. We therefore look forward to and encourage studies including formal ancestral state reconstructions to facilitate the comparison of rates between systems, using methods such as the one proposed above.

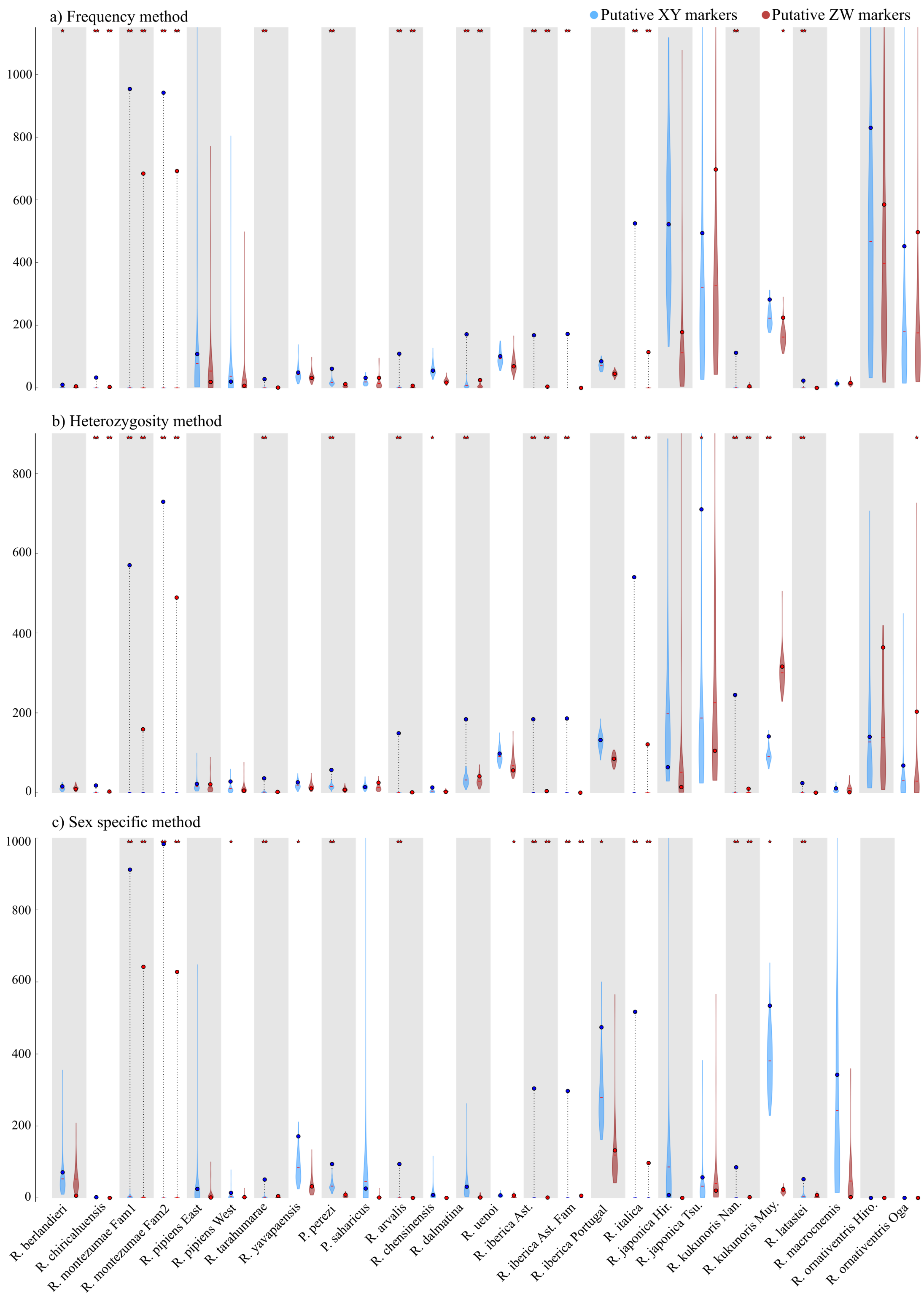

Supplementary Figure 1. Validation of the sex linked marker identification. Violin plots show the number of sex linked markers identified when using 1000 randomised male and female assignments across samples and the points show the number of sex linked markers identified using the correct sex assignments. Blue and red colors represent the marker sets supporting XY and ZW systems respectively. Red stars indicate whether the number of sex linked markers identified using the real sex assignments was above the 95 percentile (1 star) or the 99th percentile (2 stars).

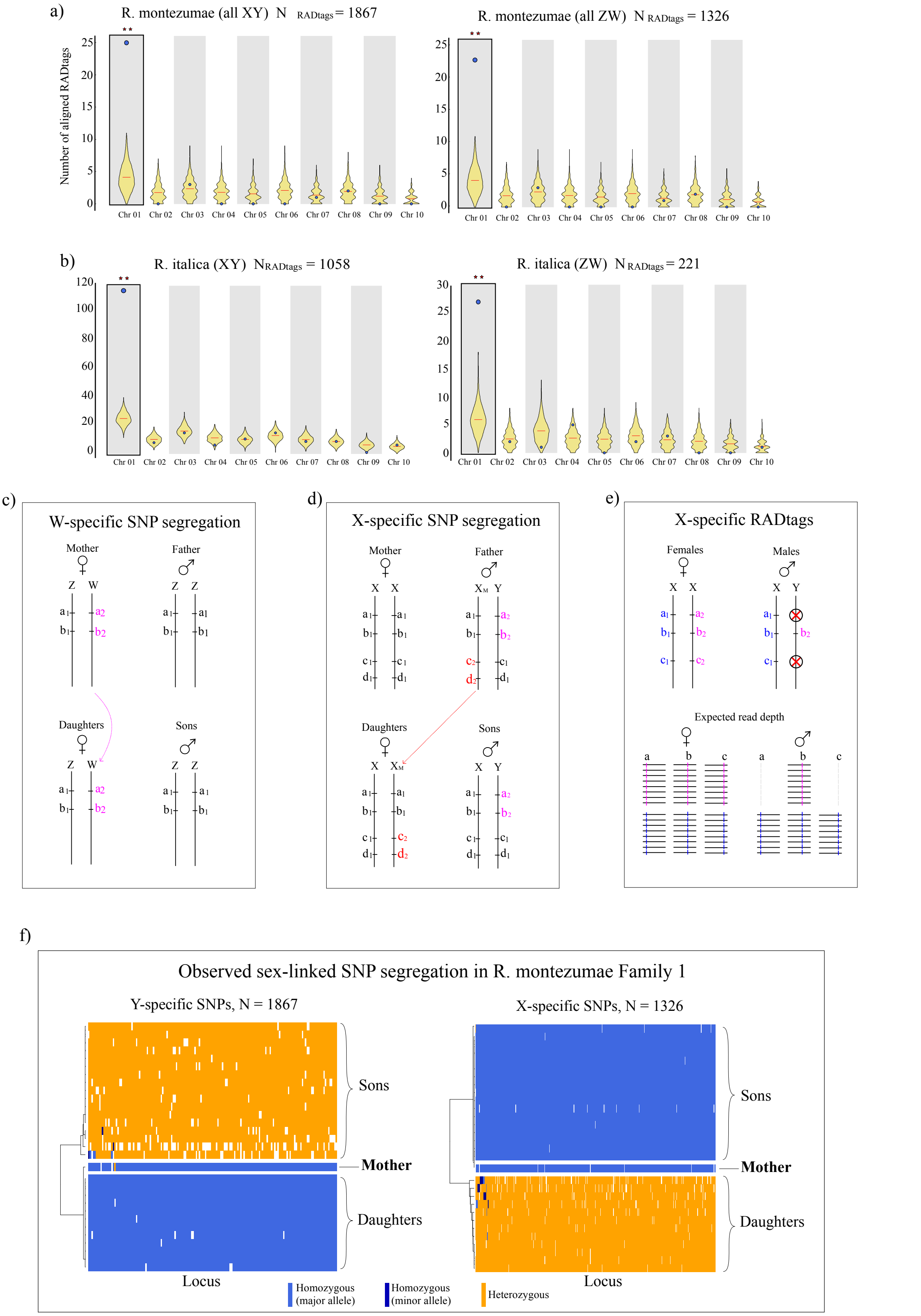

Supplementary Figure 2. Hypothesis to explain the apparent coexistence of both XY-like and ZW-like markers which map to the same chromosome. (a) Two *R. montezumae*

families and (b) a single *R. italica* family. As there is no current model of sex determination that can account for both systems on the same chromosome, we hypothesised that the ZW-like loci are in fact either loci with alleles specific to the paternal X chromosome, or X-specific markers. Importantly, we can distinguish between W-specific and paternal-X-specific SNPs by examining the genotype of the mother. If these loci truly represent a ZW system, the mother (ZW) would be heterozygous for all sex-linked SNPs which are heterozygous in daughters (c). In contrast, if these loci are in fact paternal-X-specific, the mother (XX) would be homozygous for all sex-linked SNPs heterozygous in daughters (d). We can test for X-specific RADtags, where the Y has a null allele (denoted by the circled red cross), by examining the difference in coverage at ZW-like loci between males and females (e). In (f) we show that segregation patterns in *R. montezumae* clearly favour the paternal-X-specific allele hypothesis, with the mother being homozygous for all sex-linked markers.

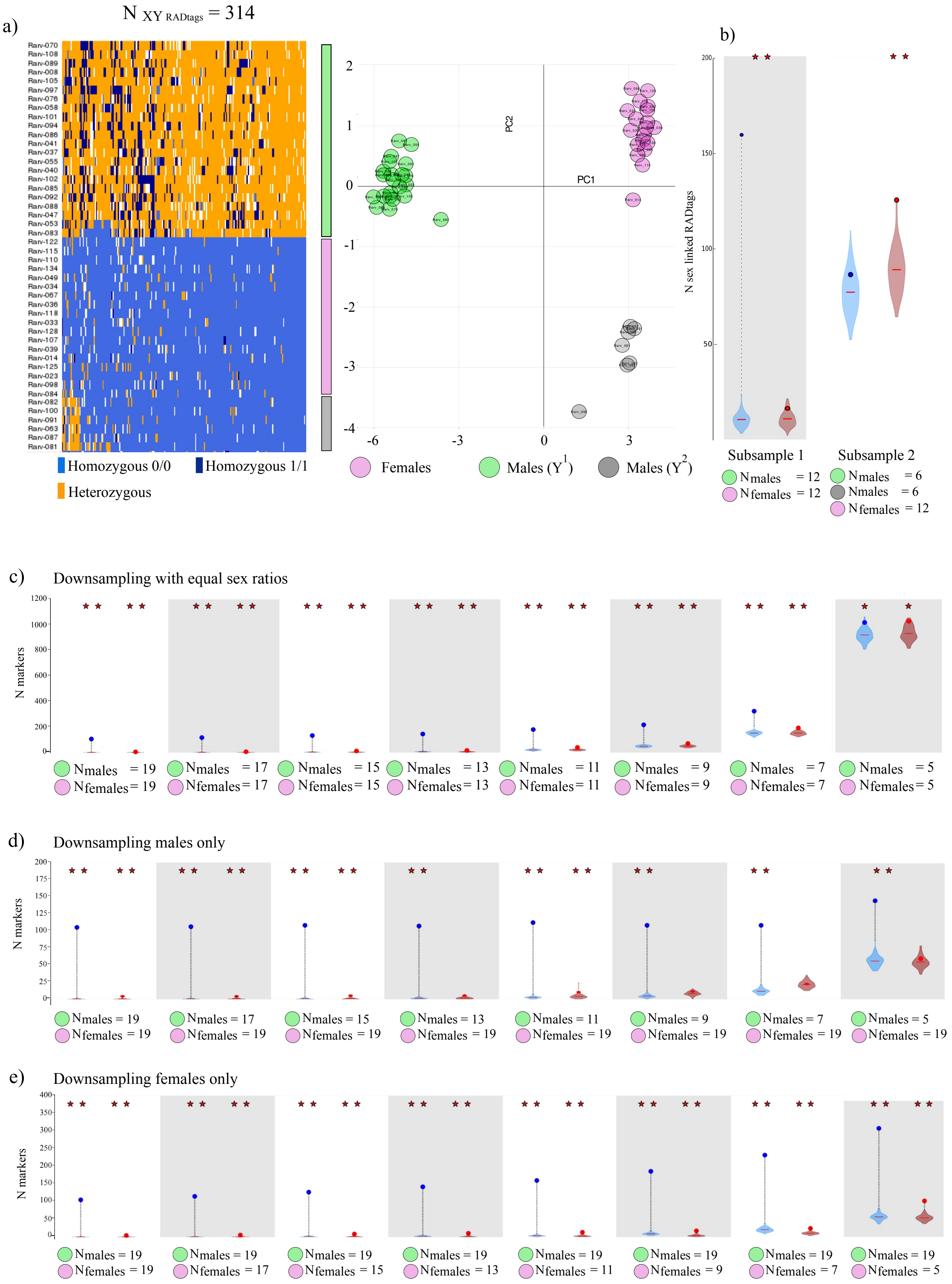

Supplementary Figure 3. **Testing which dataset parameters predict the success of sex-linked marker tests in *R. arvalis*.** a) clustering individuals by sex chromosome genotype

shows two groups of males in the sample set (from a single natural population), representing two distinct Y haplotypes. b) subsampling shows that including males from both haplotypes (subsample 2) drastically decreases success of screens for sex linkage compared to the same sample sizes containing only one Y haplotype (Subsample 1). c) As sample number is reduced (equally across sexes), false positive rates increase, reducing the likelihood that any given putatively sex-linked marker identified is truly sex linked. Skewing samples towards either females d) or males e) again results in higher false positive rates although including more males in the dataset results in higher numbers of putatively sex-linked markers identified relative to false positives. All downsampling analyese in c) – e) were performed using males from the Y^1 only.

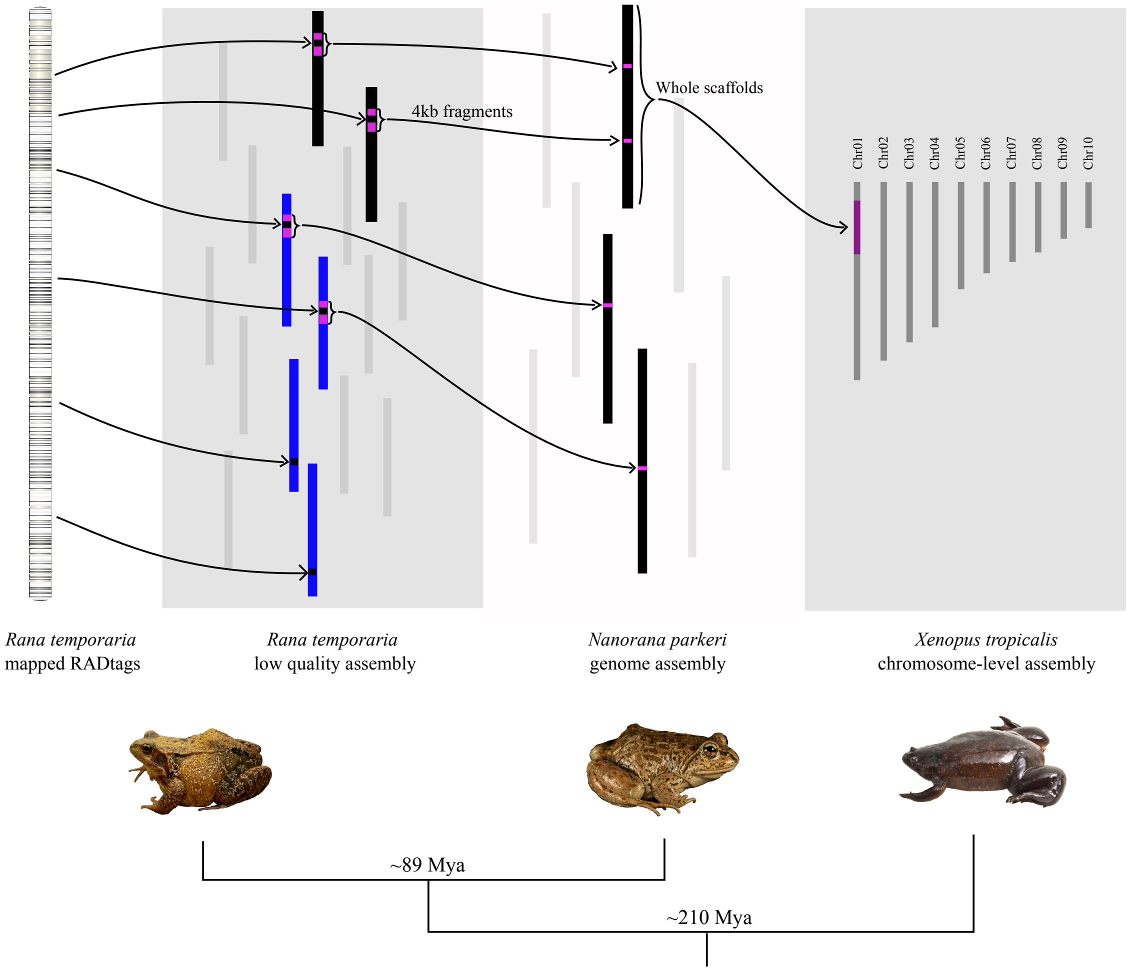

Supplementary Figure 4. Schematic describing the placement of *R. temporaria* genome scaffolds relative to the *X. tropicalis* reference chromosomes. First RADtags from the *R. temporaria* linkage maps were aligned, using blastn, to the scaffolds of the *R. temporaria* genome assembly. Next, windows up to 4kb long around each confident RADtag alignment were extracted and themselves aligned to the *N. parkeri* genome assembly. Finally *N. parkeri* scaffolds containing one or more confident alignments were aligned to the *X. tropicalis* reference genome. *R. temporaria* linkage groups could then be assigned to their respective *X. tropicalis* reference chromosomes. Importantly, placement of *R. temporaria* scaffolds with a RADtag alignment, which could not be aligned to either *N. parkeri* or *X. tropicalis* (shown in blue), was still possible based on their linkage with scaffolds which could be placed. Photo credits: *R. temporaria*: DLJ; *N. parkeri*: Yufan Wang (Amphibiachina.org); *X. tropicalis*: GL.

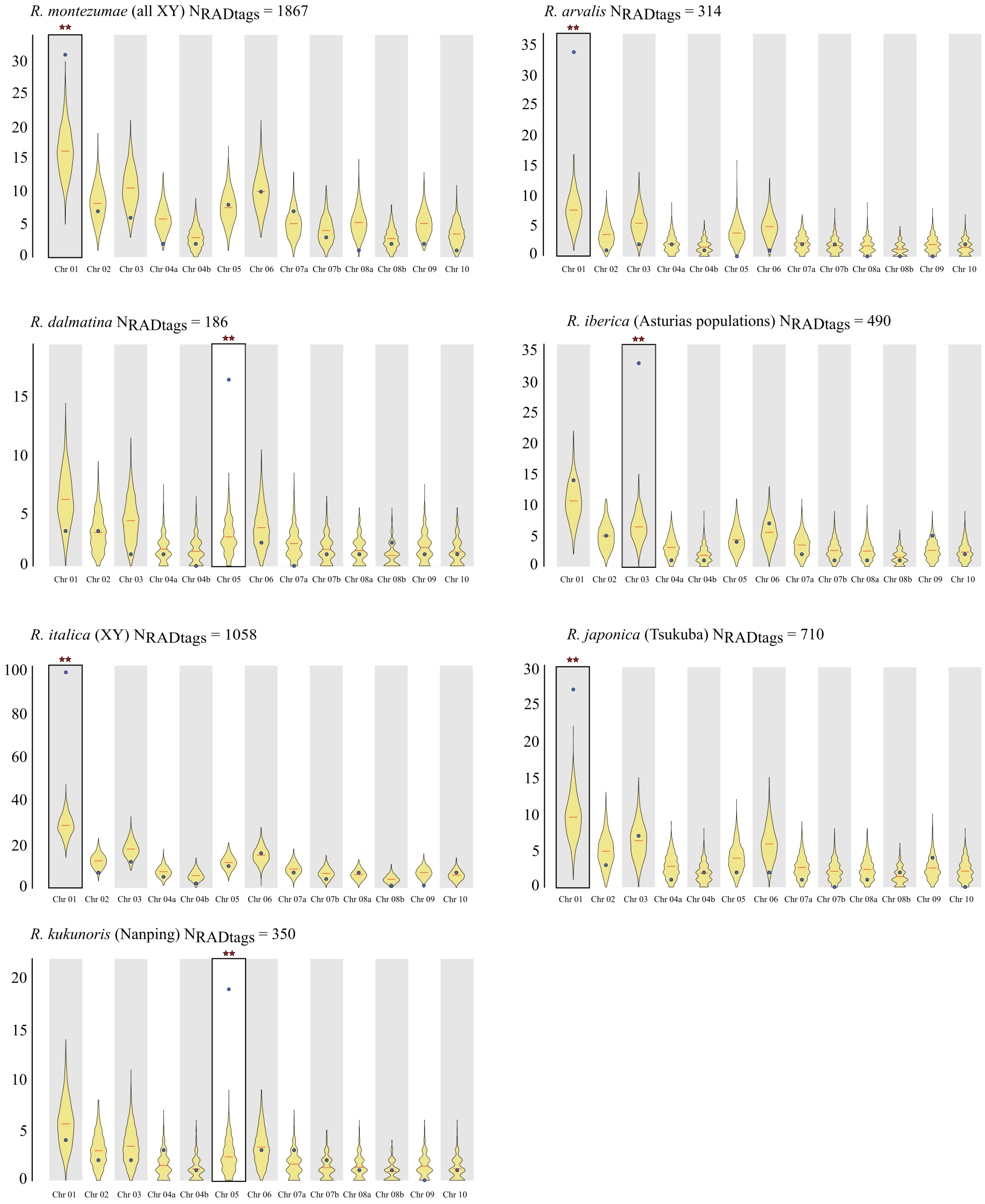

Supplementary Figure 5. Number of aligned RADtags (y axes), per reference chromosome, for 7 species of Ranidae for which a sex chromosome could be identified. Violin plots show the number of markers (mean indicated by the red line) aligned to each chromosome for each of the 1000 random subsamples of RADtags. Each random sub-sample is of the same size (N RADtags) as the number of putative sex linked markers for that species. Red stars indicate that the number of sex linked markers falls above the 99th (2 stars) percentile of the null distribution for that chromosome.

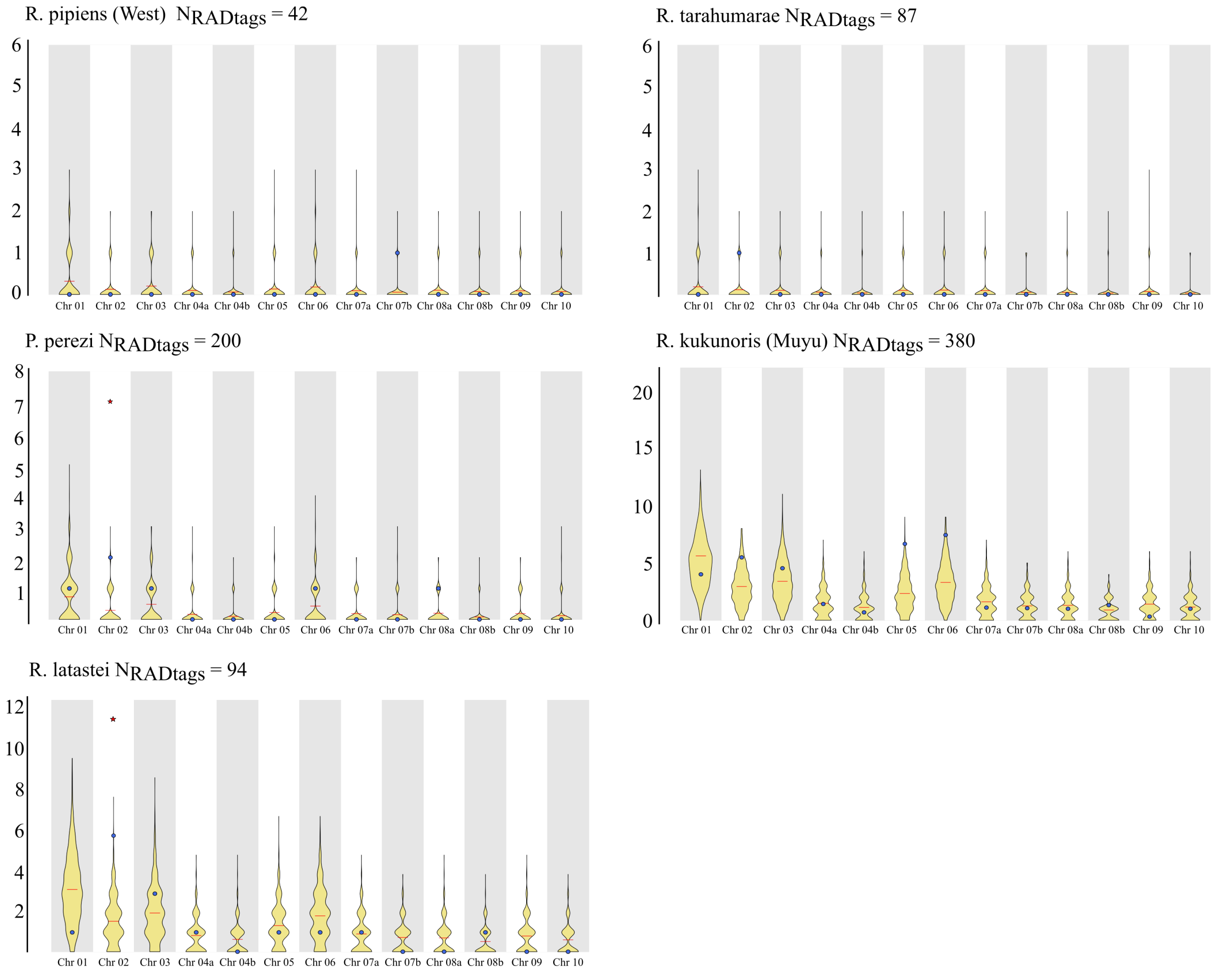

Supplementary Figure 6. Number of aligned RADtags (y axes), per reference chromosome, for 5 species of Ranidae for which a sex chromosome could not be identified due to a lack of a clear consensus from sex linked marker alignments. Violin plots show the number of markers (mean indicated by the red line) aligned to each chromosome for each of the 1000 random subsamples of RADtags. Each random sub-sample is of the same size (N RADtags) as the number of putative sex linked markers for that species. Red stars indicate that the number of sex linked markers falls above the 95th percentile of the null distribution for that chromosome.

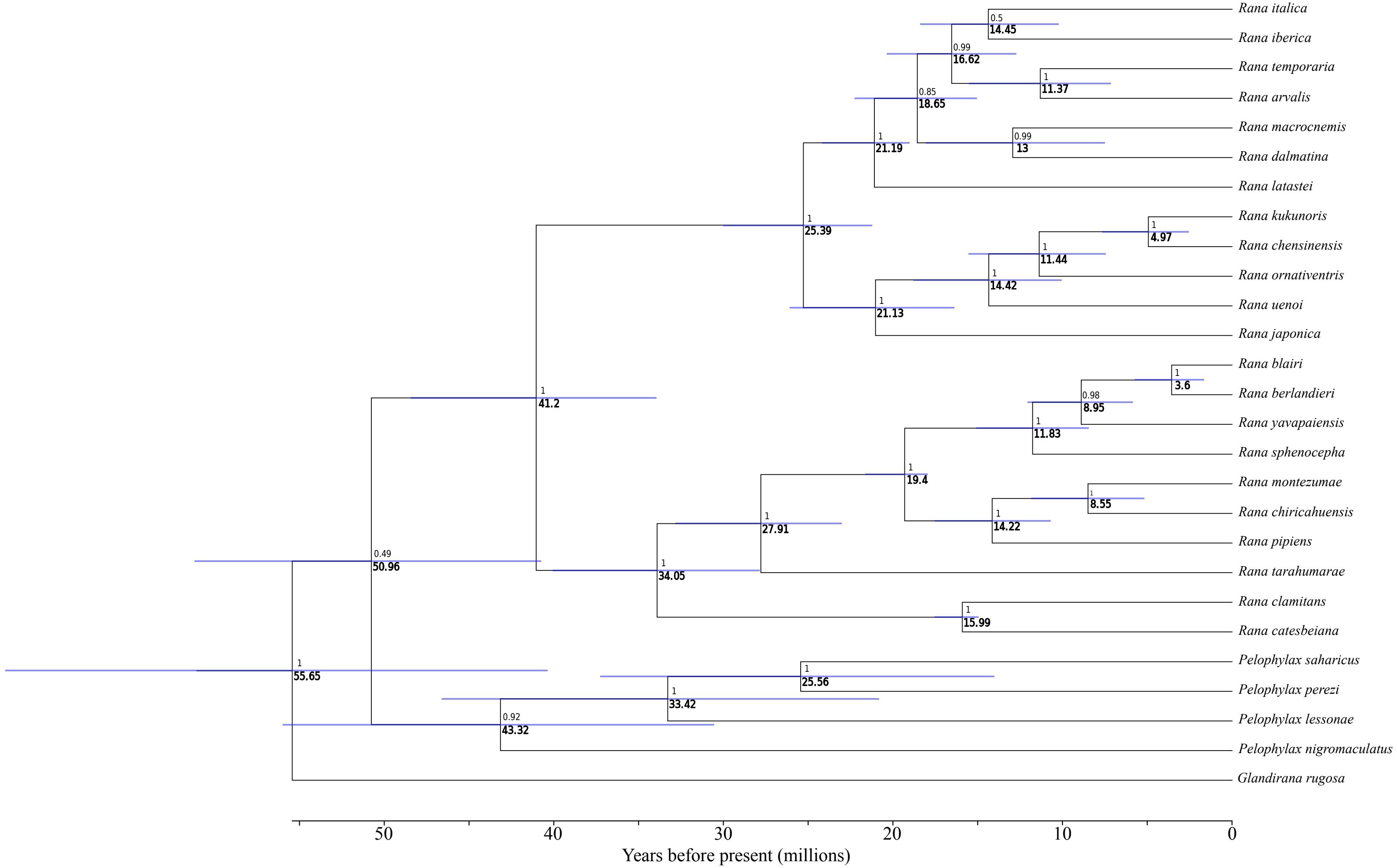

Supplementary Figure 7. Fully annotated date-calibrated phylogeny for 27 species of Ranidae. Node ages given in bold, with 95% confidence intervals represented by blue bars. Posterior support given above node ages.

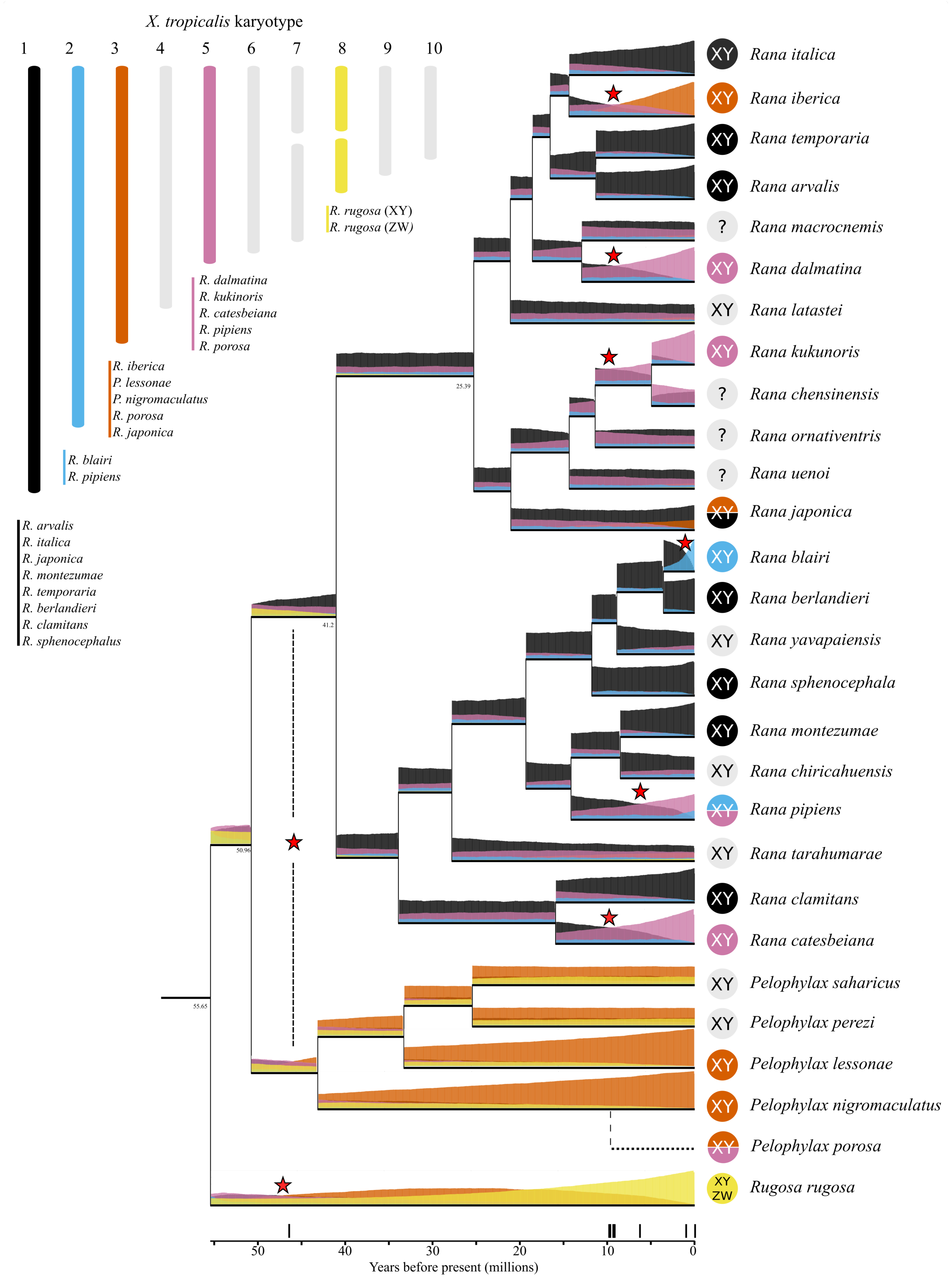

Supplementary Figure 8. Stochastic mapping estimates (using 1000 trees) of sex-chromosome states across the phylogeny. Inferred states for each tree are plotted as overlapping bar plots along each branch, thus the colour on top at each point in the tree represents the most likely state at that point. Turnovers (indicated by red stars) were inferred from positions where the most likely state switches.

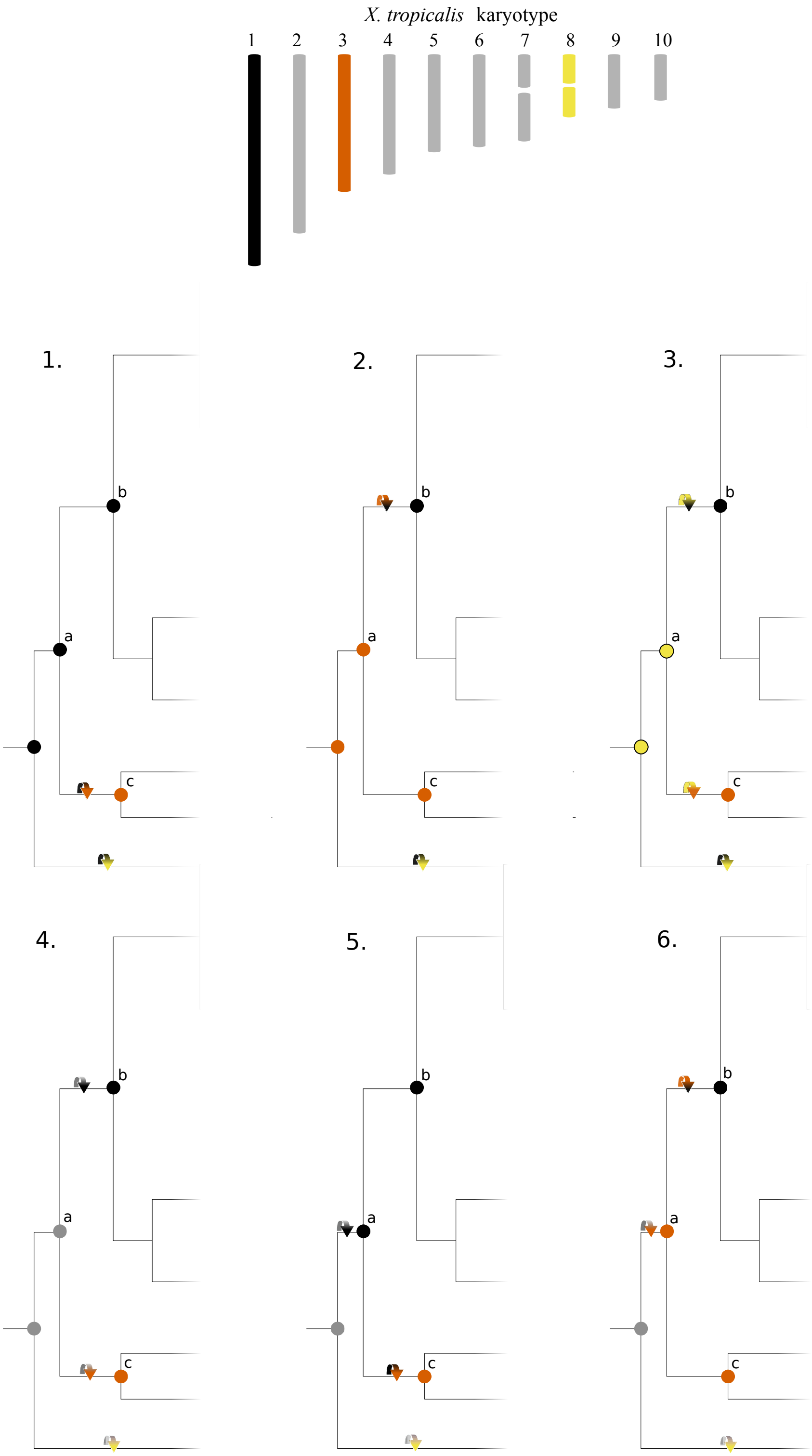

Supplementary Figure 9. Six hypotheses for the unresolved ancestral states and sex-chromosome turnover events in the deep sections of the Ranidae phylogeny. Nodes a, b and c correspond to those of the same name in Fig. 1. Grey represents any chromosome other than Chr01, Chr03 or Chr08. Hypotheses 1 and 2 are most parsimonious as they require only two turnovers, thus, these hypotheses are represented by turnover 1 on Fig. 1.

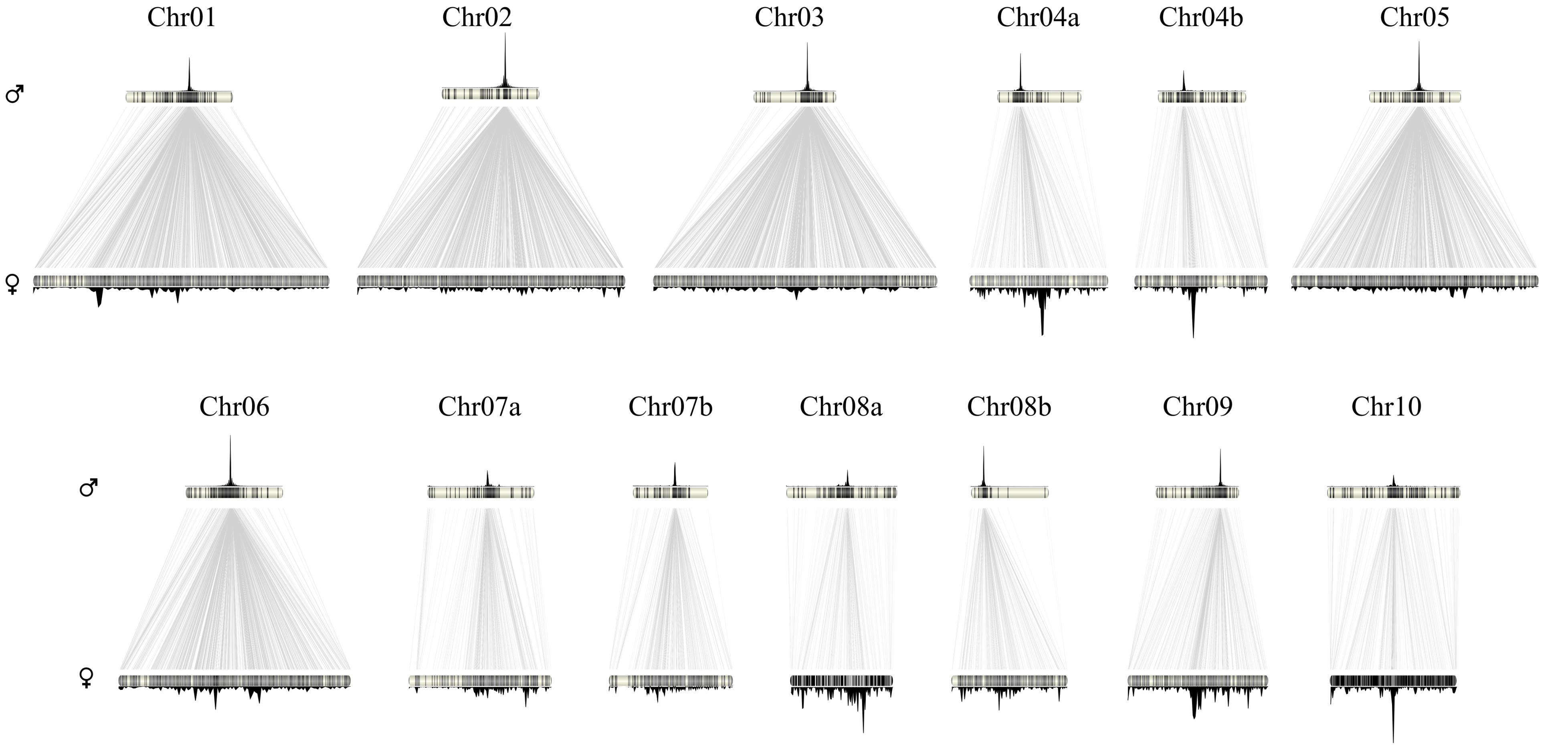

Supplementary Figure 10. Male and female linkage maps produced using 6 *R. temporaria* families from southern Sweden. In total 10,853 RADtags could be mapped and 13 linkage groups were found, corresponding to the 13 chromosome pairs in *R. temporaria*. Linkage groups are ordered here according to their *X. tropicalis* homolog (note that *X. tropicalis* has only 10 chromosome pairs). Map sizes indicate that male recombination is almost entirely absent in the centre of chromosomes. Density plots above and below each linkage group give the relative (within linkage groups) abundance of SNPs across their length.

Supplementary Table 1. Details of RAD sequencing datasets for the 20 species analysed.

Species	Population	Location		N Males	N Females	N RAD tags (N SNPs)	N sex-linked tags identified (XY / ZW)				System*	Sex chromosome**
		Lat	Long				Frequency method	Heterozygosity method	Sex specific method	Total used for mapping		
R. berlanderi		33.07	-112.36	11	9	31715 (11460)	9/4	16/8	51/6	0/0	?	?
R. chiricahuensis		32.85	-109.94	21	17	15573 (6541)	**33**/2	**18**/0	2/1	**37**/0	XY	?
R. montezumae	Fam1	19.32	-99.20	12	19	86172 (19871)	**954/684**	**570/159**	**912/642**	**1867/1326**	XY	Chr 01
	Fam2	19.32	-99.20	17	20	89951 (24076)	**942/692**	**729/489**	**983/628**	**1925/1320**	XY	Chr 01
R. pipiens	Combined			41	48	43894 (64329)	0/0	0/0	0/0	0/0	?	?
	East	-66.104 – -89.858	-44.7 – -46.92	12	16	66482 (41106)	108/19	22/21	25/1	0/0	?	?
	West	-93.537 – -107.289	42.419 – 46.952	26	20	77961 (47320)	17/7	**28**/5	**14**/2	**42**/0	XY	?
R. sphenocephala		25.41 – 33.13	80.5 – 85.21	13	15	146854 (62154)	8/4	10/5	0/0	0/0	?	?
R. tarahumarae		33.21	-114.21	8	12	50693 (2351)	**28**/1	**36**/2	**51**/5	**88**/0	XY	?
R. yavapaensis		33.71	-111.71	9	10	56731 (42934)	49/32	26/10	171/32	0/0	?	?
P. perezi		40.85	3.64	15	9	63168 (34826)	**61**/11	**57**/6	**94**/9	**200**/0	XY	?
P. saharicus		28.95	-9.93	14	7	45469 (11256)	23/32	14/25	26/1	0/0	?	?
R. arvalis		47.10	16.50	29	19	60789 (57559)	**109**/7	**149**/1	**107**/0	**314**/0	XY	Chr 01
R. chensinensis		32.64	105.40	16	11	43041 (24356)	55/13	6/2	8/0	0/0	?	?
R. dalmatina		46.51	6.37	12	7	106661 (12198)	**171**/24	**184**/37	31/1	**186**/0	XY	Chr 05
R. uenoi		35.79	127.14	8	10	67621 (71462)	101/69	98/56	7/0	0/0	?	?
R. iberica	Asturias	43.42	-5.04	35	30	74419 (3566)	**168**/4	**184**/4	**304**/1	**490**/0	XY	Chr 03
	Asturias family	43.42	-5.04	35	29	69340 (3149)	**172/0**	**186/0**	**297**/6	**483**/0	XY	Chr 03
	Central Portugal	40.50	-8.21	10	6	67062 (33126)	85/45	132/85	474/132	0/0	?	?
R. italica	Family	42.26	12.06	29	30	56465 (3676)	**525/114**	**540/121**	**517/97**	**1058/221**	XY	Chr 01
R. japonica				11	9	37455(61975)	9/33	16/94	9/1	34/0	?	?
	Hiroshima - West	34.68	133.20	6	5	82136 (44181)	522/178	64/14	8/0	0/0	?	?
	Tsukuba – East	36.08	140.08	5	5	56243 (39036)	494/697	**710**/105	57/20	**710**/0	XY	Chr 01
R. kukunoris	Nanping_corrected	33.24	104.25	13	25	47292 (30597)	**112**/5	**245**/10	**85**/2	**350**/0	XY	Chr 05
	Muyu_Banqiao	32.64	105.40	5	12	51133 (40076)	**282**/224	**141**/316	534/24	**380**/0	XY	?
R. latastei		45.85	8.96	14	14	37038 (3212)	**36**/0	**36**/0	**52**/8	**94**/0	XY	?
R. macrocnemis				6	8	5251 (3577)	14/16	11/1	342/2	0/0	?	?
R. ornativentris	Hirosaki	40.53	140.45	5	5	69074 (36996)	830/585	140/364	0/0	0/0	?	?
	Oga	39.93	139.76	6	6	52041(56707)	452/497	68/**203**	0/0	0/**203**	?	?
				362	374							
				736								

* Only assigned if sex-linked markers passed the permutation tests

** Only assigned if sex-linked markers passed the random mapping tests

Where more than one population was grouped, latitude and longitude are given as ranges encompassing all sampling sites

Bold font indicates sets of putatively sex-linked markers which passed tests permutation tests for high false positive rates and were thus used for alignment to the reference genome.

Supplementary Table 2. Results of MSTmap and LepMap3 (Combined map) linkage mapping for 6 *R. temporaria* families and their alignment to *R. temporaria, N. parkeri* and *X. tropicalis* genome assemblies.

Family	N offspring	N loci in linkage map	*R. temporaria* scaffolds anchored to linkage map	*N. parkeri* scaffolds	*N.parkeri* scaffolds mapped to a *X. tropicals* chromosome
1	42	3731	2063	946	309
2	47	4004	2218	965	303
3	57	2937	1580	681	216
4	61	3617	1972	938	287
5	54	4166	2226	991	330
6	79	4444	2408	1052	337
**Total (unique)**	340	**15313**	**12467**	**4208**	**1782**
**Combined map**	340	10853	5372	1113	378

Supplementary Table 3. Results of literature search for sex determination system and sex chromosome identity for Ranid species.

Species	System	*X. tropicalis* chromosome	Evidence	Reference
Rana temporaria				
most populations	XY	1	sex-linked microsatellite markers, DMRT1 polymorphism	36, 37, 19
Ammarnäs	XY	1 and 2	linkage map	38
Cossonay	none	none	linkage map with sufficient marker density to reject linkage of any genomic region with sex	15
Rana arvalis	XY	1	Sex-linked RADseq markers	9
Rana japonica				
most populations	XY	1	linkage with Ab	35
Ichinoseki population	XY	3	linkage with MPI	35
Akita population	?	?	absence of linkage with the 11 tested enzymes	35
Rana blairi	XY	2	linkage with SOD-1	30
Rana berlandieri	XY	1	linkage with Alb, F16DP, ADH, PGM1, β-Glu	29
Rana sphenocephala	XY	1	linkage with ACON-1	29
Rana pipiens				
population 1	XY	2	linkage with PEP-C, SOD-1	29
population 2	XY	5	linkage with ME-1	29
Rana clamitans	XY	1	linkage with ACON-1	32
Rana catesbeiana	XY	5	linkage with LDH-B	31
Pelophylax lessonae	XY	3	linkage with MPI, LDH-B, HK-1, PEP-B	33
Pelophylax nigromaculatus				
most populations	XY	3	linkage with MPI, SORDH, ENO, HK	34
some males of Kaika population	?	?	absence of linkage with the 4 tested enzymes	34
Pelophylax porosa (brevipoda)				
Konko population	XY	3	linkage with MPI, LDH-B, PEP-B	34
Maibara population	XY	5	linkage with ME-1	34
Glandirana rugosa				
most populations	XY	8	linkage with AAT	39,40
ZW-group	ZW	8	linkage with AAT	39,40
Neo ZW-group	ZW	8	linkage with AAT	40

Note: Undetected system or sex chromosomes are designated by a '?' whereas established absence of system or sex chromosome are designated by 'none'.
Numbered references correspond to supplementary reference list

Supplementary Table 4. Genbank accession numbers for all gene sequences used to produce the phylogeny for our focal species.

	Mitochondrial			Nuclear					
**Yuan et al 2016**	**12S-16S**	**CYTB**	**ND2**	**RAG1**	**RAG2**	**BDNF**	**SLC8A3**	**TYR**	**POMC**
Rana arvalis	KX269197	KX269344	KX269413	KX269562	KX269637	KX269272	KX269714	KX269789	KX269486
Rana berlandieri	AY779235	KX269301	KX269370	KX269515	KX269591	KX269226	KX269666	KX269742	KX269439
Rana blairi	AY779237								
Rana catesbeiana	KX269208	KX269354	KX269423	KX269573	KX269648	KX269283	KX269725	DQ360044	KX269497
Rana chensinensis	KX269186	KX269333	KX269402	KX269551	KX269626	KX269261	KX269703	KX269779	KX269475
Rana chiricahuensis	AY779225	KX269303	KX269372	KX269517		KX269228	KX269668	KX269744	KX269441
Rana clamitans	AY779204	KX269304	KX269373	KX269518	KX269593	KX269229	KX269669	KX269745	KX269442
Rana dalmatina	KX269198			KX269563	KX269638	KX269273	KX269715	KX269790	KX269487
Rana uenoi	KX269188	KX269335	KX269404	KX269553	KX269628	KX269263	KX269705	KX269781	KX269477
Rana iberica	KX269195	KX269342	KX269411	KX269560	KX269635	KX269270	KX269712	KX269787	KX269484
Rana japonica	KX269220	KX269364	KX269434	KX269585	KX269660	KX269295	KX269736	KX269811	KX269509
Rana kukunoris	KX269185	KX269332	KX269401	KX269550	KX269625	KX269260	KX269702	KX269778	KX269474
Rana latastei	AY147946	AY147967							
Rana macrocnemis	KX269194	KX269341	KX269410	KX269559	KX269634	KX269269	KX269711	KX269786	KX269483
Rana montezumae	AY779223	KX269309	KX269379	KX269525	KX269600	KX269236	KX269676	KX269752	KX269449
Rana ornativentris	KX269187	KX269334	KX269403	KX269552	KX269627	KX269262	KX269704	KX269780	KX269476
Rana pipiens	AY779221								
Rana sphenocephala	AY779251	KX269321	KX269391	KX269538	KX269614	KX269249	KX269690	KX269766	KX269463
Rana tarahumarae	AY779218	KX269322	KX269392	KX269539	KX269615	KX269250	KX269691	KX269767	KX269464
Rana temporaria	KX269196	KX269343	KX269412	KX269561	KX269636	KX269271	KX269713	KX269788	KX269485
Rana yavapaiensis	AY779240	KX269319		KX269535	KX269611	KX269246	KX269687	KX269763	KX269460
Pelophylax nigromaculatus	KX269216	KX269361	KX269431	KX269581	KX269656	KX269291	KX269732	KX269807	KX269505

**Pyron & Wiens 2011**	**12S**	**16S**	**CYTB**
Rana italica	AY043044	AY147945	EU595501
Rana pipiens	DQ347039	Y10945	EU370725
Pelophylax lessonae	AB023395	AY147982	AY057100
Pelophylax perezi	AY332763	AY147985	DQ902146
Pelophylax porosus	AB036401	---	AB029939
Pelophylax saharicus	AY332765	AY147984	DQ902147
Glandirana rugosa	AB430352	AB430352	AF205093

Supplementary Table 5. Gene and codon partition models recommended by PartitionFinder 2 analyses.

Partition	Best Model	# sites	Genes / codons							
1	GTR+I+G	858	cytb_1	tRNA_Ala_2						
2	GTR+I+G	2031	12S_1	tRNA_Trp_3	tRNA_Ala_1	ND2_3	tRNA_Ala_3	16S_1		
3	HKY+I+G	1591	RAG_1_1	TYR_1	BDNF_1	RAG_2_1	TYR_2	SLC8M3_1	POMC_A_2	RAG_2_2
4	TVM+I+G	802	ND2_1	TYR_3	POMC_A_3	POMC_A_1	BDNF_3	tRNA_Asn_3	tRNA_Asn_2	
5	HKY+I+G	945	tRNA_Trp_1	tRNA_Asn_1	RAG_1_2	SLC8M3_2	BDNF_2			
6	K81UF+G	914	RAG_2_3	SLC8M3_3	tRNA_Trp_2	RAG_1_3				
7	GTR+I+G	254	ND2_2							

Supplementary References

1. Gamble, T. & Zarkower, D. Identification of sex-specific molecular markers using restriction site-associated DNA sequencing. *Mol. Ecol. Resour.* **14,** 902–913 (2014).
2. Gamble, T. *et al.* Restriction Site-Associated DNA Sequencing (RAD-seq) reveals an extraordinary number of transitions among gecko sex-determining systems. *Mol. Biol. Evol.* **32,** 1296–1309 (2015).
3. Lambert, M. R., Skelly, D. K. & Ezaz, T. Sex-linked markers in the North American green frog (*Rana clamitans*) developed using DArTseq provide early insight into sex chromosome evolution. *BMC Genomics* **17,** 844 (2016).
4. Kafkas, S., Khodaeiaminjan, M., Güney, M. & Kafkas, E. Identification of sex-linked SNP markers using RAD sequencing suggests ZW/ZZ sex determination in Pistacia vera L. *BMC Genomics* **16,** 98 (2015).
5. Fowler, B. L. S. & Buonaccorsi, V. P. Genomic characterization of sex-identification markers in Sebastes carnatus and Sebastes chrysomelas rockfishes. *Mol. Ecol.* **25,** 2165–2175 (2016).
6. Gamble, T. Using RAD-seq to recognize sex-specific markers and sex chromosome systems. *Mol. Ecol.* **25,** 2114–2116 (2016).
7. Rodrigues, N., Betto-Colliard, C., Jourdan-Pineau, H. & Perrin, N. Within-population polymorphism of sex-determination systems in the common frog (*Rana temporaria*). *J. Evol. Biol.* **26,** 1569–1577 (2013).
8. Rodrigues, N., Merilä, J., Patrelle, C. & Perrin, N. Geographic variation in sex-chromosome differentiation in the common frog (*Rana temporaria*). *Mol. Ecol.* **23,** 3409–3418 (2014).
9. Brelsford, A., Lavanchy, G., Sermier, R., Rausch, A. & Perrin, N. Identifying homomorphic sex chromosomes from wild-caught adults with limited genomic resources. *Mol. Ecol. Resour.* **17,** 752–759 (2016).
10. Berset-Brändli, L., Jaquiéry, J., Broquet, T., Ulrich, Y. & Perrin, N. Extreme heterochiasmy and nascent sex chromosomes in European tree frogs. *Proc. Biol. Sci.* **275,** 1577–1585 (2008).
11. Miura, I., Ohtani, H., Kashiwagi, A., Hanada, H. & Nakamura, M. Structural differences between XX and ZW sex lampbrush chromosomes in *Rana rugosa* females (Anura: Ranidae). *Chromosoma* **105,** 237–241 (1996).
12. Brelsford, A., Dufresnes, C. & Perrin, N. High-density sex-specific linkage maps of a European tree frog (*Hyla arborea*) identify the sex chromosome without information on offspring sex. *Heredity* **116,** 177–181 (2015).
13. Brandvain, Y. & Coop, G. Scrambling eggs: meiotic drive and the evolution of female recombination rates. *Genetics* **190,** 709–723 (2012).

14. Rodrigues, N. *et al.* Dmrt1 polymorphism and sex-chromosome differentiation in Rana temporaria. *Molecular Ecology* **26,** 4897–4905 (2017).

15. Brelsford, A., Rodrigues, N. & Perrin, N. High-density linkage maps fail to detect any genetic component to sex determination in a *Rana temporaria* family. *J. Evol. Biol.* **29,** 220–225 (2016).

16. Wu, Y., Bhat, P. R., Close, T. J. & Lonardi, S. Efficient and accurate construction of genetic linkage maps from the minimum spanning tree of a graph. *PLoS Genet.* **4,** e1000212 (2008).

17. Taylor, J. *ASMap: Linkage map construction using the MSTmap algorithm*. (2016).

18. Sun, Y.-B. *et al.* Whole-genome sequence of the Tibetan frog *Nanorana parkeri* and the comparative evolution of tetrapod genomes. *Proc. Natl. Acad. Sci. U. S. A.* **112,** E1257–62 (2015).

19. Brelsford, A. *et al.* Homologous sex chromosomes in three deeply divergent anuran species. *Evolution* **67,** 2434–2440 (2013).

20. Palomar, G. *et al.* Comparative high-density linkage mapping reveals conserved genome structure but variation in levels of heterochiasmy and location of recombination cold spots in the common frog. *G3* **7,** 637–645 (2017).

21. Rastas, P., Paulin, L., Hanski, I., Lehtonen, R. & Auvinen, P. Lep-MAP: fast and accurate linkage map construction for large SNP datasets. *Bioinformatics* **29**, 3128-3134 (2013).

22. Altschul, S. F., Gish, W., Miller, W., Myers, E. W. & Lipman, D. J. Basic local alignment search tool. *J. Mol. Biol.* **215,** 403–410 (1990).

23. Kumar, S., Stecher, G., Suleski, M. & Hedges, S. B. TimeTree: A resource for timelines, timetrees, and divergence times. *Mol. Biol. Evol.* **34,** 1812–1819 (2017).

24. Sumida, M. & Ogata, M. Intraspecific differentiation in the japanese brown frog *Rana japonica* inferred from mitochondrial DNA sequences of the cytochrome b gene. *Zoolog. Sci.* **15,** 989–1000 (1998).

25. Sutherland, B. J. G., Rico, C., Audet, C. & Bernatchez, L. Sex chromosome evolution, heterochiasmy and physiological QTL in the salmonid brook charr *Salvelinus fontinalis*. *G3* (2017). doi:10.1534/g3.117.040915

26. Myosho, T., Takehana, Y., Hamaguchi, S. & Sakaizumi, M. Turnover of sex chromosomes in celebensis group medaka fishes. *G3* **5,** 2685–2691 (2015).

27. Setiamarga, D. H. E. *et al.* Divergence time of the two regional medaka populations in Japan as a new time scale for comparative genomics of vertebrates. *Biol. Lett.* **5,** 812–816 (2009).

28. Mokodongan, D. F. & Yamahira, K. Origin and intra-island diversification of Sulawesi endemic Adrianichthyidae. *Mol. Phylogenet. Evol.* **93,** 150–160 (2015).

29. Wright, D. A. & Richards, C. M. Linkage groups in the leopard frog, *Rana pipiens*. in *Genetic Maps: Locus Maps of Complex Genomes* (ed. O'Brien, S. J.) 4326–4329 (Cold Spring Harbor Laboratory Press, 1993).

30. Wright, D. A. & Richards, C. M. Two sex-linked loci in the leopard frog, *Rana pipiens*. *Genetics* **103,** 249–261 (1983).

31. Elinson, R. P. Genetic analysis of developmental arrest in an amphibian hybrid (*Rana catesbeiana*, *Rana clamitans*). *Dev. Biol.* **81,** 167–176 (1981).

32. Elinson, R. P. Inheritance and expression of a sex-linked enzyme in the frog, *Rana clamitans*. *Biochem. Genet.* **21,** 435–442 (1983).

33. Hotz, H., Uzzell, T. & Berger, L. Linkage groups of protein-coding genes in western palearctic water frogs reveal extensive evolutionary conservation. *Genetics* **147,** 255–270 (1997).

34. Nishioka, M. & Sumida, M. The position of sex-determining genes in the chromosomes of *Rana nigromaculata* and *Rana brevipoda*. *Sci. Rep. Lab. Amphibian Biol. Hiroshima Univ.* **13,** 51–97 (1994).

35. Sumida, M. & Nishioka, M. Geographic variability of sex-linked loci in the Japanese brown frog, *Rana japonica*. *Sci. Rep. Lab. Amphibian Biol. Hiroshima Univ.* **13,** 173–195 (1994).

36. Ma, W.-J., Rodrigues, N., Sermier, R., Brelsford, A. & Perrin, N. Dmrt1 polymorphism covaries with sex-determination patterns in *Rana temporaria*. *Ecol. Evol.* **6,** 5107–5117 (2016).

37. Cano, J. M., Li, M.-H., Laurila, A., Vilkki, J. & Merilä, J. First-generation linkage map for the common frog *Rana temporaria* reveals sex-linkage group. *Heredity* **107,** 530–536 (2011).

38. Rodrigues, N., Vuille, Y., Brelsford, A., Merilä, J. & Perrin, N. The genetic contribution to sex determination and number of sex chromosomes vary among populations of common frogs (*Rana temporaria*). *Heredity* **117,** 25–32 (2016).

39. Miura, I., Ohtani, H., Nakamura, M., Ichikawa, Y. & Saitoh, K. The origin and differentiation of the heteromorphic sex chromosomes Z, W, X, and Y in the frog *Rana rugosa*, inferred from the sequences of a sex-linked gene, ADP/ATP translocase. *Mol. Biol. Evol.* **15,** 1612–1619 (1998).

40. Ogata, M., Hasegawa, Y., Ohtani, H., Mineyama, M. & Miura, I. The ZZ/ZW sex-determining mechanism originated twice and independently during evolution of the frog, *Rana rugosa*. *Heredity* **100,** 92–99 (2008).
